# *Saccharomyces cerevisiae*–Based Platform for Rapid Production and Evaluation of Eukaryotic Nutrient Transporters and Transceptors for Biochemical Studies and Crystallography

**DOI:** 10.1371/journal.pone.0076851

**Published:** 2013-10-04

**Authors:** Peter Scharff-Poulsen, Per Amstrup Pedersen

**Affiliations:** 1 Department of Biology, University of Copenhagen, Copenhagen, Denmark; 2 Center for Microbial Biotechnology, Department of Systems Biology, Technical University of Denmark, Kongens Lyngby, Denmark; University of South Florida College of Medicine, United States of America

## Abstract

To produce large quantities of high quality eukaryotic membrane proteins in *Saccharomyces cerevisiae*, we modified a high-copy vector to express membrane proteins C-terminally-fused to a Tobacco Etch Virus (TEV) protease detachable Green Fluorescent Protein (GFP)-8His tag, which facilitates localization, quantification, quality control, and purification. Using this expression system we examined the production of a human glucose transceptor and 11 nutrient transporters and transceptors from *S. cerevisiae* that have not previously been overexpressed in *S. cerevisiae* and purified. Whole-cell GFP-fluorescence showed that induction of GFP-fusion synthesis from a galactose-inducible promoter at 15°C resulted in stable accumulation of the fusions in the plasma membrane and in intracellular membranes. Expression levels of the 12 fusions estimated by GFP-fluorescence were in the range of 0.4 mg to 1.7 mg transporter pr. liter cell culture. A detergent screen showed that n-dodecyl-ß-D-maltopyranoside (DDM) is acceptable for solubilization of the membrane-integrated fusions. Extracts of solubilized membranes were prepared with this detergent and used for purifications by Ni-NTA affinity chromatography, which yielded partially purified full-length fusions. Most of the fusions were readily cleaved at a TEV protease site between the membrane protein and the GFP-8His tag. Using the yeast oligopeptide transporter Ptr2 as an example, we further demonstrate that almost pure transporters, free of the GFP-8His tag, can be achieved by TEV protease cleavage followed by reverse immobilized metal-affinity chromatography. The quality of the GFP-fusions was analysed by fluorescence size-exclusion chromatography. Membranes solubilized in DDM resulted in preparations containing aggregated fusions. However, 9 of the fusions solubilized in DDM in presence of cholesteryl hemisuccinate and specific substrates, yielded monodisperse preparations with only minor amounts of aggregated membrane proteins. In conclusion, we developed a new effective *S. cerevisiae* expression system that may be used for production of high-quality eukaryotic membrane proteins for functional and structural analysis.

## Introduction

Nutrient transporters are the gatekeepers controlling transport of essential nutrients such as sugars and amino acids across the plasma membrane of cells. From a medical and a pharmaceutical perspective, human nutrient transporters are of great importance because (i) various gene defects in nutrient transporters have been identified and shown to cause human diseases, (ii) nutrient transporters are potential drug targets, (iii) drugs are transported into cells using nutrient transporters (as reviewed by [1], see also Genomic Transporter Database of SLC (Solute Carrier) gene tables at web-site http://www.pharmaconference.org/slctable.asp). Obviously, structural information about nutrient transporters is of great interest to both academia and the pharmaceutical industry. Nevertheless, structures are only known for a few nutrient transporters of bacterial origin, whereas structures of eukaryotic transporters are not yet available.

Nutrient transporters from yeast constitute straight-forward targets for gaining valuable insight into structure-function relationships of similar transporters from higher eukaryotic organisms. In yeast, sugars and amino acids are transported across the plasma membrane by transporters of the major facilitator superfamily (MFS) and of the amino acid-polyamine-organocation (APC) superfamily. As shown in the TransportDB database (http://www.membranetransport.org/) the yeast MFS comprises 85 members, of which 20 have functions in hexose transport, and the yeast APC family comprises 24 members, of which 18 have functions in amino acid transport.

An interesting aspect of the nutrient transporters from yeast is the finding that some of them also have receptor functions involved in signal transduction processes (reviewed in [2]). These so-called “transceptors” constitute a novel concept in signaling, and comprise both transporting and non-transporting transceptors. Non-transporting transceptors include the glucose sensors Snf3 and Rgt2 (reviewed in [3]) and the amino acid sensor Ssy1 (reviewed in [4]). Transporting transceptors include the Gap1 amino acid transporter [5], [6], the Pho84 phosphate transporter [7] and the Mep2 ammonium transporter [8]. Growing evidence for transporters functioning as transceptors in humans, fruit flies and plants, suggests that transceptors are widespread in nature and that we may only have recognized the tip of the iceberg [2], [9].

Recent studies of the amino acid transceptor Ssy1 and the glucose transceptor Snf3 from yeast [10], [11], [12], [13] revealed that Ssy1 and Snf3 are able to sense both extracellular and intracellular nutrients, and a mechanistic model that explains how these transceptors may participate in maintaining intracellular homeostasis for nutrients in yeast cells was proposed. This model may be of importance for understanding how for instance pertubations in amino acid availability affect growth of cancer cells, and assist in identification of new anti-cancer targets and development of cancer treatment therapies [14]. Similarly, the results obtained with Snf3 may add to the understanding of the human glucose transceptor GLUT2 [15] which has been proposed to be involved in regulation of food intake by the hypothalamus [16].

Members of the Proton-dependent Oligopeptide Transporter (POT) family, which include the *S. cerevisiae* Ptr2 oligopeptide transporter, are also of great interest in nutrient uptake. In addition to their function in peptide transport in organs such as the gastrointestinal tract, the kidney and the central nervous system, they are also responsible for uptake of a large variety of pharmaceuticals with peptide-like structures such as penicillins, antivirals and anticancer agents [17].

Although the yeast nutrient transporters and transceptors have high physiological importance and implications for the function of similar mammalian proteins they have not yet been purified and characterized biochemically and structurally. Therefore, to deepen our understanding of eukaryotic nutrient transporters and transceptors, we here report on the production of a selection of these membrane proteins using a high-copy vector expression system [18] combined with the GFP-fusion methodology developed by Drew et al 2008 [19]. We show that reasonable amounts of transporters and transceptors can be produced in a high quality. With these results, we may provide biochemical characterizations and make the first moves towards crystallization and structure determination of this class of eukaryotic membrane proteins, with significant perspectives in physiology and drug development.

## Materials and Methods

### Yeast strains and culture conditions

Overexpression of membrane protein-GFP fusions in *S. cerevisiae* was performed in strain PAP1500 (*MAT*α *ura3*-*52 trp1:: GAL10-GAL4 lys2*-*801 leu2*Δ*1 his3*Δ*200 pep4::HIS3 prb1*Δ*1.6R can1* GAL*)* [18] as follows: PAP1500 transformed with various expression constructs was inoculated in 5 ml of synthetic minimal (SD) media [20], [21] supplemented with glucose (20 g/L), leucine (60 mg/L) and lysine (30 mg/L) and incubated over night at 30°C with shaking. The over-night culture was diluted 50 times in SD media supplemented with lysine (30 mg/L) and incubated for 2 days at 30°C with shaking to increase plasmid copy number during leucine-limitation. Next, the expression strain was cultivated in SD media supplemented with glucose (5 g/L), glycerol (3% v/v), alanine (20 mg/L), arginine (20 mg/L), aspartic acid (100 mg/L), cysteine (20 mg/L), glutamic acid (100 mg/L), histidine (20 mg/L), lysine (30 mg/L), methionine (20 mg/L), phenylalanine (50 mg/L), proline (20 mg/L), serine (375 mg/L), threonine (200 mg/L), tryptophan (20 mg/L), tyrosine (30 mg/L), and valine (150 mg/L). Thus, 1 liter of this medium was inoculated with the leucine-starved culture to give an OD_450_ of 0.05 and cultivation was continued at 30°C with shaking until OD_450_ reached 1.0. Cultures were thermo-equilibrated to 15°C (unless otherwise stated) and induction of GFP-fusion synthesis was initiated with addition of 110 ml 20% (w/v) galactose dissolved in the above described medium lacking glucose. Incubation at 15°C was continued with shaking for 48 hours. Cells were harvested by centrifugation at 3,000 rpm in a SLA-3000 rotor (Sorvall) for 5 minutes at 5°C. A complete list of strains used is available in Table S1.

### Construction of expression plasmids

Transporter and transceptor genes were PCR amplified from chromosomal DNA with AccuPol DNA polymerase (Ampliqon DK) and primers (TAG Copenhagen) shown in Table S2. Each GFP-8His expression plasmid was generated by *in vivo* homologous recombination in *S. cerevisiae* by transforming strain PAP1500 with a transporter PCR fragment, a GFP PCR fragment and *Bam*HI and *Hin*dIII digested pEMBLyex4 [22]. A TEV site (GENLYFQSQF) was introduced between the transporter and the GFP tag. GFP was C-terminally fused to a sequence of 8 His moieties. PAP1500 transformants were selected on SD plates with leucine (60 mg/L) and lysine (30 mg/L). All plasmid constructs were checked by DNA sequencing at Eurofins MWG Operon (Germany).

### Membrane preparation

Small scale preparation of crude yeast membranes was carried out by a glass bead disruption method. A cell pellet from a 1 liter culture (usually 3 g wet weight) was resuspended in 10 ml ice-cold lysis buffer (25 mM imidazole adjusted to pH 7.5 with HCl, 10% sucrose (w/v), 1 mM EDTA, 1 mM EGTA, and protease inhibitors (1 mM PMSF, leupeptin (1 µg/ml), pepstatin (1 µg/ml) and chymostatin (1 µg/ml)). Approximately 10 ml of glass beads (425 to 600 microns, Sigma) were added to the cell suspension. Cells were disrupted by 4 times 1 minute high speed whirlimixing interrupted by 1 minute of cooling on ice. The supernatant was collected and glassbeads were washed two times in 10 ml ice-cold lysisbuffer. Unbroken cells and cell debris were removed from the combined supernatants by centrifugation at 10,000 rpm in a SS-34 rotor (Sorvall) for 10 minutes at 5°C. This method typically disrupted 85% of the PAP1500 cells as measured by GFP fluorescence. Membranes were collected by centrifugation of the final supernatant at 40,000 rpm in a 70 TI rotor (Beckman) for 1.5 hour at 5°C. Membrane pellets were resuspended in 3 ml buffer (20 mM phosphate pH 7.0, 200 mM NaCl, 10% glycerol, 10 mM imidazole, 1 mM PMSF and 1 µg/ml of leupeptin, pepstatin and chymostatin, respectively) using a rotating Potter-type homogenizer.

### Protein quantification

The protein concentration in crude membranes was determined by the Lowry assay [23]. The concentration of proteins in purified fractions was measured using a Nanodrop apparatus (Thermo Scientific).

### Bioimaging of live yeast cells

*S. cerevisiae* cells used for bioimaging were grown and induced for production of GFP-fusions as described [24]. Fluorescence was visualized at 1,000 x magnification with a Nikon Eclipse E600 fluorescence microscope equipped with an Optronics Magnafire model S99802 camera.

### Whole-cell fluorescence

Five ml of yeast culture with a known optical density was harvested by centrifugation at 3000 rpm for 3 minutes at 5°C in a Multifuge 3 S-R (Heraus). The supernatant was carefully removed and cells were re-suspended in 100 µl sterile water and transferred to a Nucleon Nunc white micro plate (Cat. No. 136101). Fluorescence was measured in a microplate spectrofluorometer (Fluoroskan Ascent, Thermo Labsystems) using 485 nm excitation and 520 nm emission.

### In-gel fluorescence

In-gel fluorescence was carried out on an Image Station 4000 MM (Carestream) using 465 nm excitation and 535 nm emission or an ImageQuant LAS 4010 imager (GE Healthcare).

### Detergent Screen

180 μl membrane extract from yeast cells overexpressing the indicated membrane protein-GFP fusions diluted in solubilization buffer (20 mM phosphate pH 7.0, 200 mM NaCl, 10% glycerol, 1 mM PMSF and 1 µg/ml of leupeptin, pepstatin and chymostatin, respectively) to a concentration of 3.5 mg protein/ml was mixed with 20 µl detergent (100 mg/ml) yielding a protein to detergent ratio of 1:3 (mg/ml). The mixture was incubated at 4°C for 1 hour with mild agitation. Non-solubilized material was pelleted by centrifugation in an Airfuge (Beckman) fitted with an A-100/30 rotor for 12 minutes at 92,000 rpm at 30 psi air pressure (167,000 g). The supernatant was transferred to a Nucleon Nunc white microplate and GFP fluorescence was measured in a Fluoroskan Ascent spectrofluorometer (Thermo Labsystems) using 485 nm excitation and 520 nm emission. Solubilization efficiency was estimated by the ratio of GFP fluorescence of the supernatant and of the detergent-solubilized membranes before centrifugation.

Solubilization of membranes in DDM and cholesteryl hemisuccinate (CHS) was carried out as described above using a 20 μl mixture of DDM (100 mg/ml) and of CHS (20 mg/ml), and addition of substrates to a final concentration of: 100 mM glucose for the Hxt1-, Hxt2-, Hxt3-, Hxt4-, Rgt2-, Snf3- and GLUT2-GFP fusions; 10 mM glutamine for the Agp1-GFP fusion; 2 mM leucine for the Ssy1- and Tat1-GFP fusions; 10 mM Gly-Leu dipeptide for the Ptr2-GFP fusion; 10 mM NH_4_Cl for the Mep2-GFP fusion.

Anagrade quality detergents and CHS were purchased from Affymetrix: DM, n-decyl-β-D-maltopyranoside; DDM, n-dodecyl-β -D-maltopyranoside; OG, n-octyl-β -D-glucopyranoside; CHAPS, 3-[(3-cholamidopropyl)-dimethylammonio]-1-propane sulfonate/N,N-dimethyl-3-sulfo-N-[3-[[3α,5β,7α,12α)-3,7,12-trihydroxy-24-oxocholan-24-yl]amino]propyl]-1-propanaminium hydroxide; CYMAL-5, 5-cyclohexyl-1-pentyl-β-D-maltoside; FC-12, n-dodecylphosphocholine.

### Fluorescence-detection size-exclusion chromatography

Fluorescence-detection size-exclusion chromatography (FSEC) was carried out essentially as described [25]. Total membranes were solubilized as described in the detergent screen section and loaded onto a Superose 12 10/300 column (GE Healthcare Life Science) mounted on an ÄKTA purifier (GE Healthcare Life Science). The column was pre-equilibrated with SEC buffer (20 mM Na^+^-phosphate pH 7.0, 200 mM NaCl, and 0.3 mg/ml DDM) and run with a flow rate of 0.5 ml/min. After 5 ml elution, fractions of 0.2 ml were collected and GFP fluorescence was measured in a Fluoroskan Ascent spectrofluorometer (Thermo Labsystems) using 485 nm excitation and 520 nm emission.

A high molecular weight gel filtration calibration kit (GE Healthcare Life Science) was used for determination of the void volume and molecular weight estimations.

### Purification of transporter-GFP fusions by Ni-NTA affinity purification

Crude membranes isolated from a 1-liter induced culture were diluted to a concentration of 3.5 mg protein/ml in lysis buffer (20 mM phosphate pH 7.0, 200 mM NaCl, 10% glycerol, 10 mM imidazole, 1 mM PMSF and 1 µg/ml of leupeptin, pepstatin and chymostatin, respectively). DDM solution (100 mg/ml) was added to a final concentration of 10 mg/ml, yielding a protein to DDM ratio of approximately 1:3. Solubilization was carried out for 1 hour at 4°C with mild agitation.

Insoluble material was removed by centrifugation at 40,000 rpm and 4°C for 1.5 hours in a Sorvall T-845 rotor. Fluorescence was measured before and after centrifugation to measure solubilization efficiency.

Solubilized membranes were incubated over night with 0.5 ml Ni-NTA Superflow resin (Qiagen) for binding of the transporter-GFP fusions in a glass beaker at 4°C with magnetic stirring. The resin was transferred to a 2 ml CellThru disposable column (Clontech) and washed in 40 column volumes of washing buffer (20 mM phosphate pH 7.0, 200 mM NaCl, 10% glycerol, 50 mM imidazol, and 0.3 mg/ml DDM). The fusion proteins were then eluted with elution buffer (20 mM phosphate pH 7.0, 200 mM NaCl, 10% glycerol, 450 mM imidazol, and 0.3 mg/ml DDM). Fluorescence was measured throughout the purification procedure to monitor the presence of fusion protein.

### GFP purification and quantification

Yeast-enhanced GFP-8His was produced in *E. coli* BL21(DE3)pLysS from plasmid pET20bGFP-8His (a generous gift from Dr. David Drew, Imperial College London, England). Histidine-tagged GFP was purified using Supplementary Protocol 2 in [26].

A set of concentrations of the purified GFP-8His was used to establish a correlation between fluorescence and the amount of GFP. The correlation was used to quantify the amount of transporter-GFP fusion in whole cells and cell extracts [19].

### TEV protease purification

*E. coli* strain BL21(DE3) Codon Plus was transformed with MBP-TEV-His protease expression plasmid (obtained from Dr. David Drew) to yield PAP8350. TEV protease was produced as follows (essentially as described in a protocol for TEV purification obtained from Dr. David Drew). Strain PAP8350 was grown at 30°C in LB medium containing 100 µg/ml ampicillin and 50 µg/ml chloramphenicol. At OD_600_  =  0.6 the culture was transferred to 25°C and IPTG was added to a final concentration of 0.4 mM to induce synthesis of the MBP-TEV fusion, which is self-cleaved at a TEV cleavage site between MBP and TEV. After shaking for 22 hours at 25°C, cells were harvested by centrifugation. Cells were re-suspended in 20 ml of 20 mM phosphate pH 7.0, 200 mM NaCl, 20% glycerol and disrupted by sonication on a Bandelin-Sonopuls 3100 apparatus for 15 minutes (Pulse on 5 seconds, Pulse off 5 seconds, amplitude 50%). Cell debris was removed by centrifugation at 24,000 rpm and 5°C for 10 minutes in a SS34 rotor. TEV protease was bound to Ni-NTA resin (Qiagen) which had been equilibrated in purification buffer (20 mM phosphate pH 7.0, 200 mM NaCl, 20% glycerol, 3 mM DTT) with 20 mM imidazole. The resin loaded with TEV protease was washed in 20 column volumes purification buffer with 50 mM imidazole, and the protease was eluted with purification buffer with 250 mM imidazole. Fractions containing TEV were dialyzed against (20 mM phosphate pH 7.0, 200 mM NaCl, 30% glycerol, 3 mM DTT). Aliquots of the TEV protease preparation were stored at – 80°C in the dialysis buffer with 50% glycerol.

### TEV protease digestion

GFP-fusions were cleaved with His-tagged TEV protease during dialysis over night at 4°C in Snake Skin dialysis tubes (Thermo Scientific). The dialysis buffer consisted of 20 mM phosphate pH 7.0, 200 mM NaCl and 0.03% DDM (w/v).

A fusion to TEV ratio of 1:3 was used for cleavage.

## Results

### High-copy expression vector for production of membrane protein-GFP fusions in *S. cerevisiae*

**Figure 1:**
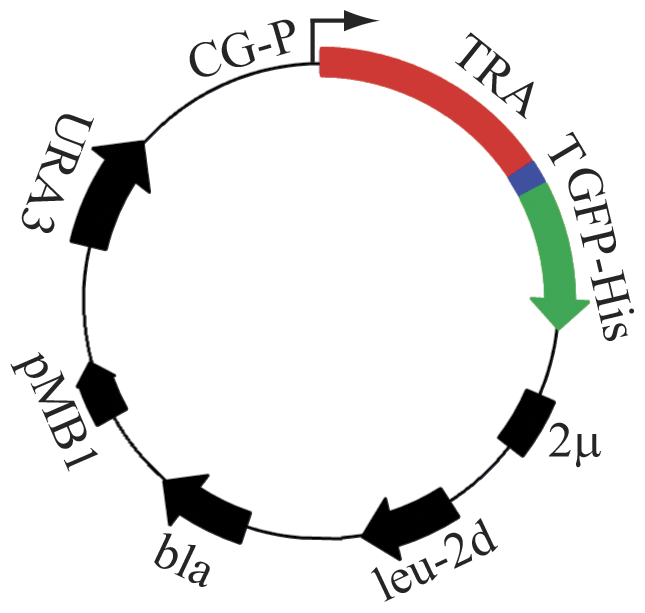
Structural map of the plasmids used for expression of membrane protein-GFP-8His fusion proteins. Abbreviations used: CG-P, a hybrid promoter carrying the *GAL10* upstream activating sequence fused to the 5’ non-translated leader of the cytochrome-1 gene; TRA, transporter/transceptor gene; T, Tobacco Etch Virus (TEV) cleavage site; GFP-His, yeast enhanced GFP cDNA fused to eight histidine codons; 2µ, the yeast 2 micron origin of replication; *leu2-d*, a poorly expressed allele of the β-isopropylmalate dehydrogenase gene; *bla*, a β-lactamase gene; pMB1, the pMB1 origin of replication; *URA3*, the yeast orotinin-5’-P decarboxylase gene. Rapid construction of the expression plasmids were carried out by insertion of transporter/transceptor and GFP PCR fragments into linearized expression vector pEMBLyex4 by *in vivo* homologous recombination in *S. cerevisiae*.

To facilitate overexpression of nutrient transporters and transceptors fused to GFP, we generated plasmid constructs as outlined in Figure 1. A collection of 12 plasmids was constructed with the following nutrient transporters and transceptors: (i) six members of the yeast MFS comprising the glucose transceptors Snf3 and Rgt2 and the hexose transporters Hxt1, Hxt2, Hxt3, and Hxt4, (ii) three yeast APC family members comprising the amino acid transceptor Ssy1 and the amino acid transporters Agp1 and Tat1, (iii) the yeast POT family peptide transporter Ptr2, (iv) the yeast Amt family ammonium transceptor Mep2 and (v) the human glucose transceptor GLUT2.

The expression vector provides: (i) a strong galactose inducible CYC-GAL promoter, which is enhanced by overexpressing the Gal4 transcriptional activator in the host strain PAP1500 [18], (ii) a poorly expressed *leu2-d* gene, which brings about a plasmid copy number in the range of 200 to 400 per haploid genome, when cells are starved for leucine [27], [28] (iii) prevention of plasmid-loss due to the presence of the *URA3* and *leu2-d* selection markers, (iv) the ability to monitor the localization, quality and quantity of fusion proteins throughout the overexpression and purification steps by fluorescence measurements. Strain PAP1500 was transformed with the various high-copy expression constructs to form the PAP1500-GFP expression system. Propagation and induction of the strains were carried out as described in Materials and Methods.

### Optimization of induction conditions for production of transporter- and transceptor-GFP fusions

**Figure 2:**
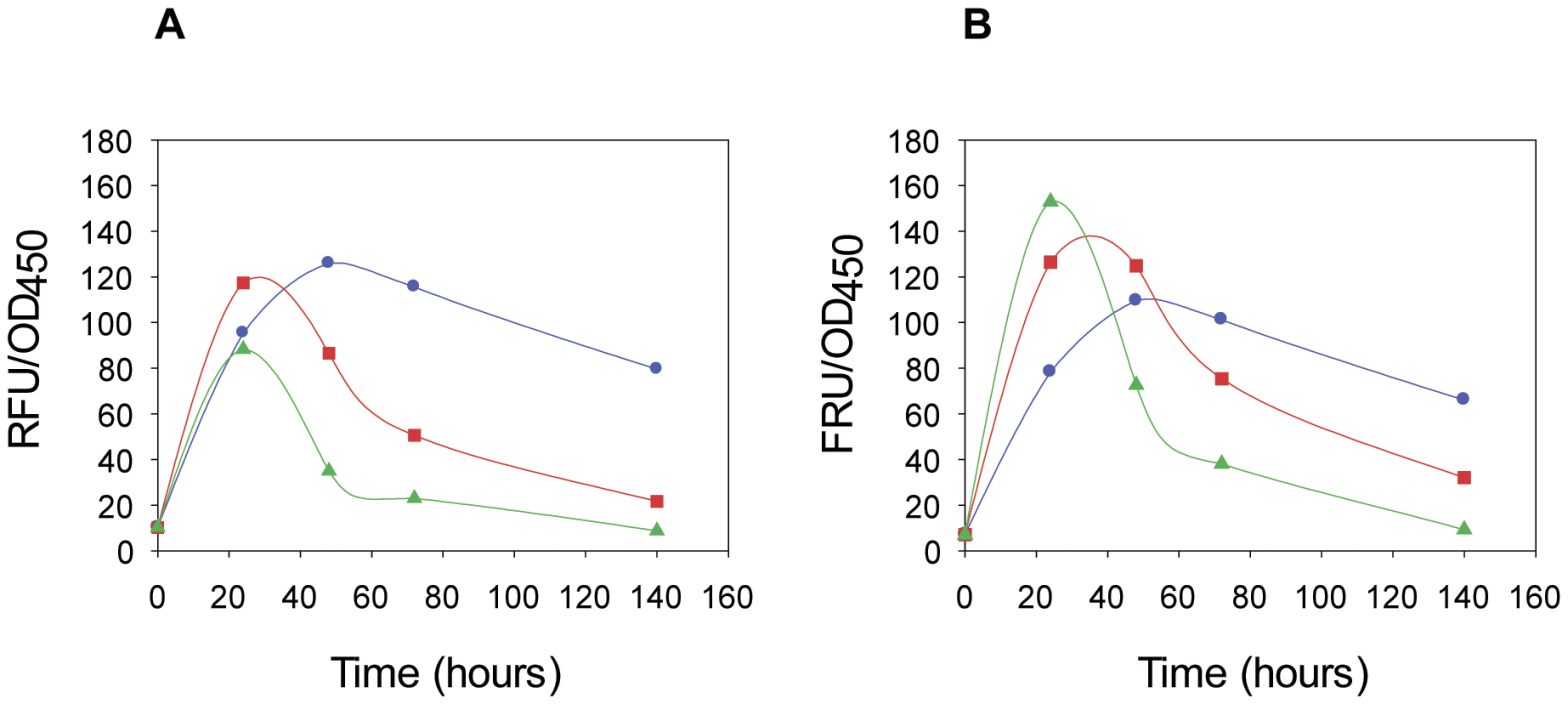
Time course for accumulation of GFP-fusions after galactose induction. Expression of Ssy1-GFP-fusions (A) and of Agp1-GFP-fusions (B) was induced by addition of galactose (T = 0) to the cultures incubated at 15°C, blue line (circles), 20°C, red line (squares) or 25°C, green line (triangles), respectively. Samples were withdrawn after 24, 48, 72 and 140 hours of induction and whole-cell GFP fluorescence and OD_450_ were measured. Relative fluorescence (RFU) divided by the cell density at OD_450_ was plotted against the time of induction. Conditions for propagation and induction of the PAP1500 host transformed with the expression constructs were carried out as described in Materials and Methods. In short, cells were depleted for leucine for several generations in SD minimal medium to increase plasmid copy-number. Cultures were subsequently propagated in minimal medium supplemented with all amino acids except leucine and isoleucine at 30°C until OD_450_ reached 1.0. Cultures were then thermo-equilibrated at 15°C, 20°C or 25°C before induction with galactose.

To explore the optimal induction time and temperature for overexpression of GFP-fusions produced in shake cultures, we measured whole-cell fluorescence to monitor accumulation of the Agp1-GFP and Ssy1-GFP fusions during induction. Figure 2A shows that accumulation of the Ssy1-GFP fusion had an optimum after induction for 48 hours at 15°C. At 20°C and 25°C an optimum was found after 24 hours of induction. However, the stability of the fusion was persistent for a longer period of time at 15°C than at 20°C and 25°C. The Agp1-GFP fusion had an optimum of accumulation after 24 hours of induction at 25°C, between 24 to 48 hours at 20°C and 48 hours at 15°C. Similarly to the Ssy1-GFP fusion, accumulation of the Agp1-GFP fusion was more stable at 15°C than at the higher temperatures. Due to these results and our experience with production of other membrane proteins, which accumulate GFP-fusions even after 300 hours of induction at 15°C, we decided to carry out expression screening of the 12 transporter and transceptor fusions using induction at 15°C for 48 hours. This induction condition will most probably ensure high production of the fusions, whereas fusions that are only transiently expressed at 20°C and 25°C may result in low production.

Finally, the induction profiles indicated that optimization of growth conditions must be carried out individually for each fusion protein to obtain the highest possible expression level, at a later stage when production of the fusions is carried out in large fermentors.

### Localization, quantity, quality and integrity of the overexpressed transporter- and transceptor-GFP fusions

**Figure 3:**
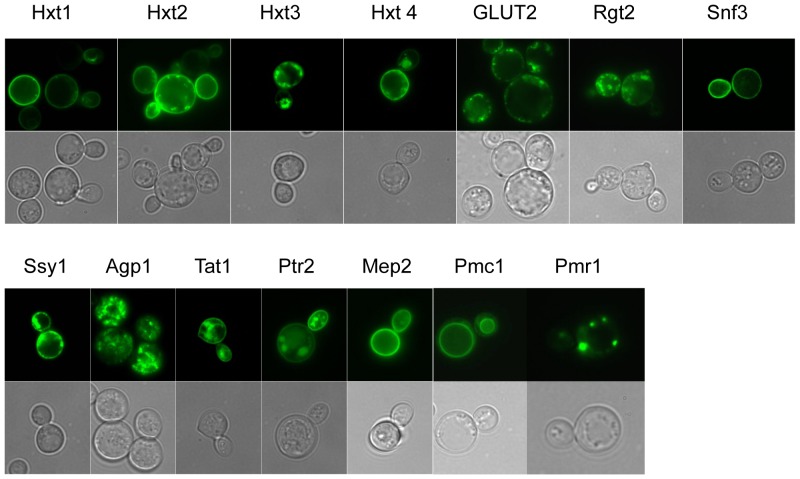
Imaging of live yeast cells expressing the various transceptor- and transporter-GFP fusions. (A) Localization of hexose transporters (Hxt1 through Hxt4) and glucose transceptors (GLUT2, Snf3, and Rgt2) accumulated in PAP1500 cells induced with galactose at 15°C for 48 hours. (B) Localization of an amino acid transceptor (Ssy1), two amino acid transporters (Agp1, Tat1), an oligopeptide transporter (Ptr2) and an ammonium ion transporter (Mep2) accumulated in PAP1500 cells induced with galactose at 15°C for 48 hours. Localization of the vacuolar Pmc1 Ca^2+^-ATPase and the Golgi located Pmr1 Ca^2+^, Mn^2+^-ATPase is included for comparison. Upper panel: GFP fluorescence. Lower panel: Phase contrast.

As a first quality assessment of the overexpressed fusions we analyzed live yeast cells by fluorescence microscopy. Since the C-terminal GFP only becomes folded and fluorescent when the upstream membrane protein integrates into the membrane, fluorescence visualizes that the fusions are membrane-integrated [19]. The results in Figure 3 show that all fusions accumulated in the plasma membrane. Although membrane-integrated expression is no guarantee of function [19] this is a promising result in the empirical process towards obtaining useful membrane proteins for functional and structural work.

In addition, accumulation in intracellular compartments can also be observed for the majority of fusions. To unravel these localization patterns we made the following analysis. Firstly, to rule out that any of the produced GFP-fusions accumulated in the vacuole and potentially are destined for degradation, we compared their localization to a GFP fusion of the Pmc1 Ca^2+^-ATPase that exclusively localizes to the vacuolar membrane [29]. As evident from Figure 3, none of the produced transporter/transceptor-GFP fusions localize to the vacuolar membrane visualized by the Pmc1-GFP fusion. To further approach the intracellular localization observed for most of the transporters, we made comparisons to bioimages of yeast producing a Pmr1-GFP fusion. Pmr1 is the secretory pathway Ca^2+^, Mn^2+^-ATPase that localizes to a Golgi compartment [30]. Since the dot-like appearance observed for Pmr1-GFP is also found for most of the transceptor/transporter GFP fusions, we suggest that the transporter fusions are also localized to Golgi.

Using the method described in [19] whole-cell GFP fluorescence was used to quantify the amount of transporter- and transceptor GFP fusions accumulated in PAP1500 cells induced with galactose at 15°C for 48 hours. The results in Table 1 show that the transporters and transceptors accumulated in the range from 0.4 to 1.7 mg per liter culture with an average yield of 0.7 mg per liter. These expression levels for 12 TM membrane proteins are quite satisfactory when compared to expression levels reported in [31], [32] and fairly good starting points for further optimization of expression. In conclusion, the quantification analysis points to a favorable overexpression potential for producing suitable amounts for biochemical and structural analysis.

**Table 1:** Yields of transporters and transceptors in whole-cells and crude membranes.

Strain	GFP-fusion	Expression in whole-cells	Expression in membranes	
		mg transporter/L	pmol transporter/mg	% of total membrane proteins
PAP7910	Ssy1-GFP-His	0.53	157	1.5
PAP8004	Agp1-GFP-His	0.60	155	1.1
PAP8143	Tat1-GFP-His	0.53	71	0.5
PAP8141	Ptr2-GFP-His	0.51	101	0.7
PAP8139	Mep2-GFP-His	0.68	148	0.8
PAP8131	Hxt1-GFP-His	0.47	67	0.4
PAP8133	Hxt2-GFP-His	0.78	126	0.8
PAP8135	Hxt3-GFP-His	0.71	84	0.5
PAP8137	Hxt4-GFP-His	0.68	126	0.8
PAP8145	Rgt2-GFP-His	1.65	98	0.8
PAP8147	Snf3-GFP-His	1.06	69	0.7
PAP7913	GLUT2-GFP-His	0.36	131	0.8

Accumulation of transporters and transceptors in expression strains induced with galactose at 15°C for 48 hours were quantified using purified yeast-enhanced GFP as a standard to correlate fluorescence to the amount of GFP protein in whole-cells and in membrane extracts as outlined [19].

The purified crude membrane fractions were found to contain 67 to 157 pmol transporter/mg membrane protein corresponding to a membrane density in the range from 0.4 to 1.5% of the total membrane protein content (Table 1). This may be appropriate for structural studies, since successful crystallization was achieved with recombinant G-protein coupled receptors (GPCRs) purified from membranes with approximately 50 pmol/mg membrane protein corresponding to 0.2% of the total membrane protein content [33], [34].

**Figure 4:**
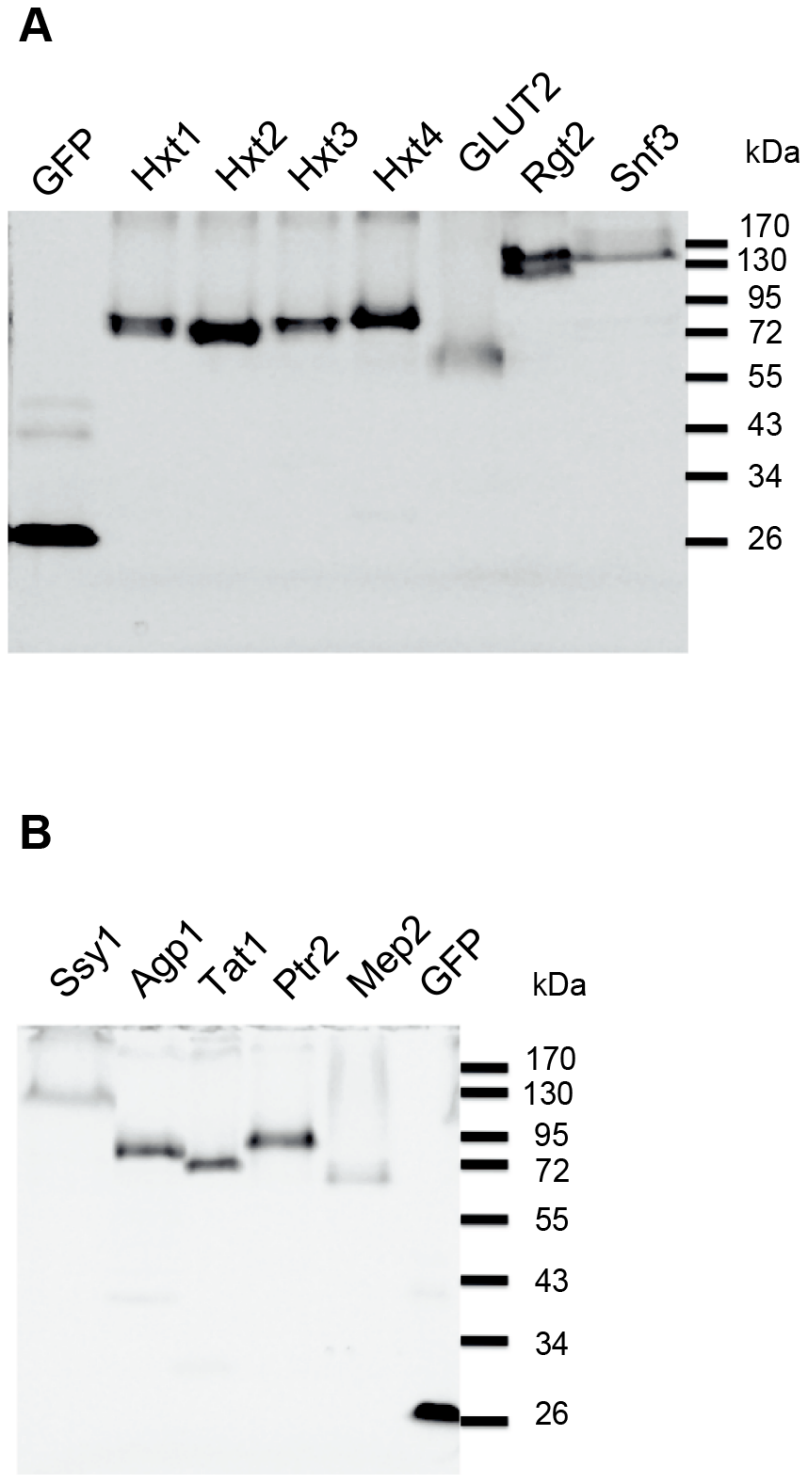
SDS-PAGE analysis of total membrane extracts from yeast cells expressing transporter-GFP fusions detected by in-gel fluorescence. (A) Analysis of Hxt1-, Hxt2-, Hxt3-, Hxt4-, GLUT2-, Rgt2- and Snf3-GFP-fusions. Membranes isolated from expression strains induced galactose for 48 hours at 15°C were subjected to SDS-PAGE and visualized by in-gel fluorescence. The calculated molecular weights of the membrane protein-GFP fusions are: Hxt1, 91 kDa; Hxt2, 88 kDa; Hxt3, 91 kDa; Hxt4, 92 kDa; Rgt2, 111 kDa; Snf3, 125 kDa; GLUT2, 86 kDa. (B) Analysis of Agp1-, Tat1-, Ptr2-, Mep2-, and Ssy1-GFP- fusions. Membranes isolated from expression strains induced with galactose for 48 hours at 15°C were subjected to SDS-PAGE and visualized by in-gel fluorescence. The calculated molecular weights of the membrane protein-GFP fusions are: Ssy1 (124 kDa), Agp1 (98 kDa), Tat1 (97 kDa), Ptr2 (96 kDa), Mep2 (81 kDa). Purified GFP (28 kDa) was also applied to the gels.

Crude membranes isolated from the expression strains were analyzed by SDS-PAGE and in-gel fluorescence to assess the quality of the recombinant transceptor- and transporter-GFP fusions. The results in Figure 4 show that all tested strains accumulated full-length GFP-fusions in accordance with their calculated molecular weights including the contribution of 10 to 15 kDa of correctly folded GFP [35]. None of the fusions show significant degradation and release of free GFP; only a negligible sign of degradation can be seen for the Hxt4-, Agp1- and Tat1-fusions. The integrity of the Ssy1-GFP fusion was also investigated by western analysis using an antibody against Ssy1. A full-length protein of 130 kDa similar to that identified by in-gel fluorescence was observed with no sign of degradation (not shown).

### Detergent screen of recombinant GFP fusions

**Figure 5:**
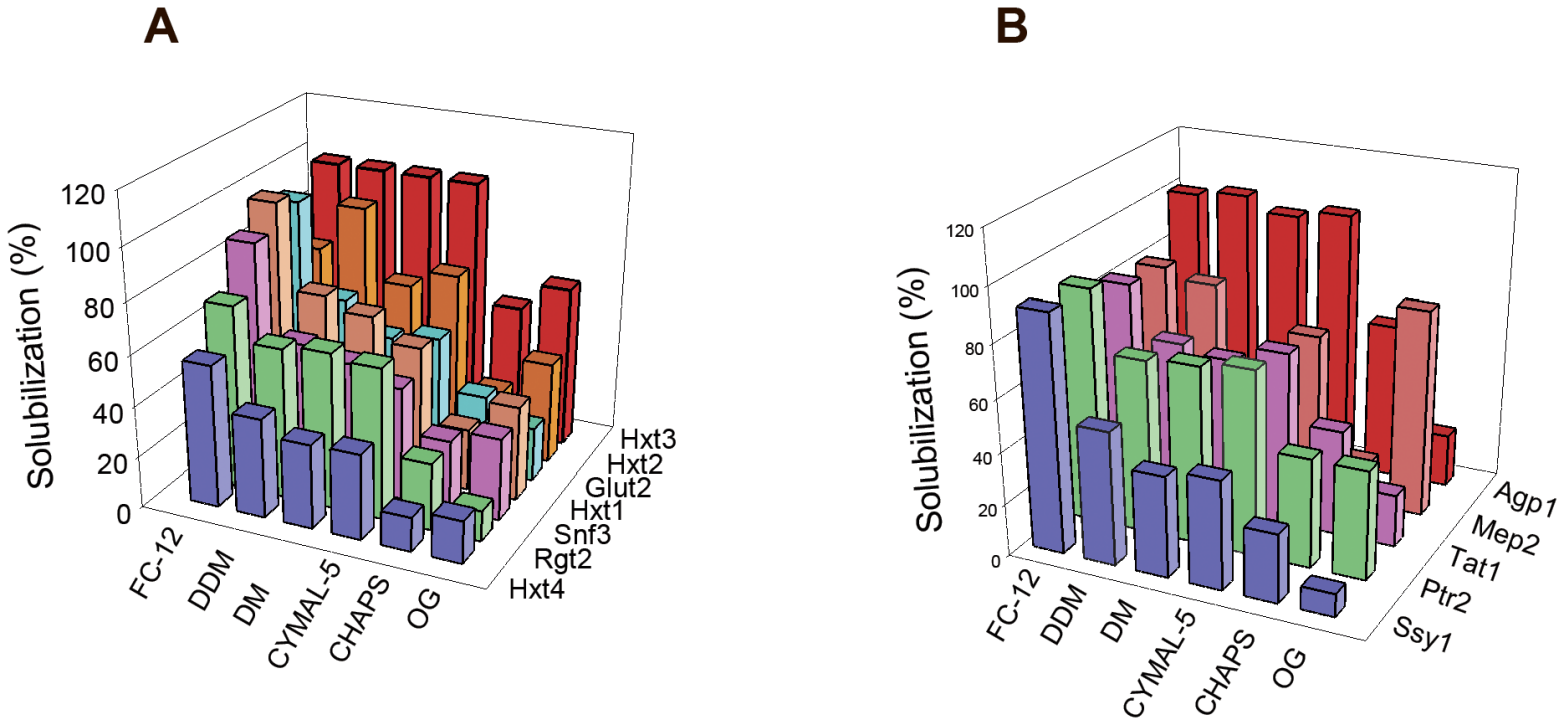
Detergent screen of transporter- and transceptor-GFP fusions. Membranes of yeast cells overexpressing the indicated (A) Hxt1-, Hxt2-, Hxt3-, Hxt4-, GLUT2-, Rgt2- and Snf3-GFP-fusions and (B) Agp1-, Tat1-, Ptr2-, Mep2-, and Ssy1-GFP- fusions were solubilized with the six detergents indicated. Detergent solubilization efficiency was estimated by GFP fluorescence of the 92,000 rpm supernatant and of the detergent-solubilized membranes before centrifugation as described in Materials and Methods.

A quick detergent screen was performed to identify appropriate detergents for solubilization of each fusion protein. The results presented in Figure 5 show that DDM, DM, FC-12 and CYMAL-5 solubilized the GFP fusions with an acceptable efficiency of about 50 to 100%, whereas OG and CHAPS resulted in poor solubilization; except for the Mep2-GFP fusion which solubilized well in OG. Since DDM is particularly compatible with TEV protease cleavage of fusion proteins (personal communication with D. Drew) we decided to use this detergent for solubilization and purification.

### Purification of transporter- and transceptor-GFP fusions by Ni-affinity chromatography

**Figure 6:**
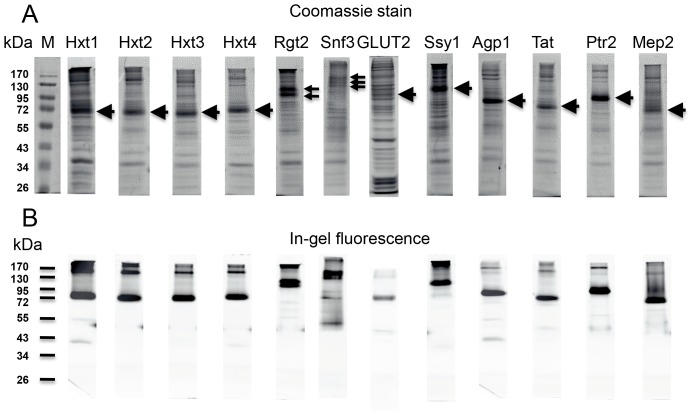
SDS-PAGE analysis of GFP-fusions isolated by Ni-NTA affinity purification. Peak-fractions from purification of Hxt1-, Hxt2-, Hxt3-, Hxt4-, Rgt2-, Snf3-, GLUT2-, Ssy1-, Agp1-, Tat1-, Ptr2-, and Mep2-GFP-fusions were subjected to SDS-PAGE and visualized by Coomassie Blue staining (upper panel)and in-gel fluorescence (lower panel). The positions of fusion proteins are indicated by arrows. Marker: PageRuler prestained protein ladder (Fermentas). The calculated molecular weights of membrane protein GFP-fusions are: Hxt1, 91 kDa; Hxt2, 88 kDa; Hxt3, 91 kDa; Hxt4, 92 kDa; Rgt2, 111 kDa; Snf3, 125 kDa; GLUT2, 86 kDa, Ssy1 (124 kDa), Agp1 (98 kDa), Tat1 (97 kDa), Ptr2 (96 kDa), Mep2 (81 kDa).

Purification of the 12 GFP-fusions included in this study was carried out once using the following procedure: Crude membranes isolated from 1-liter shake cultures of the various expression strains induced at 15°C for 48 hours were solubilized in DDM and purified by Ni-NTA chromatography using a one-step block gradient procedure as outlined in Materials and Methods. GFP fluorescence was measured throughout the purification steps to monitor the yield and the elution profile. As evident from the fluorescence measurements the binding efficiency to the Ni-NTA was on average 70% and the recovery in the elution peak was on average 50%. SDS-PAGE analysis by Coomassie Blue staining and in-gel fluorescence of the peak fractions from each purification show that partial purifications of the various fusions were accomplished (Figure 6).

Comparisons of the protein bands detected show that the intense Coomassie stained bands correspond to the fluorescent bands visible by in-gel fluorescence. In addition, the prominent protein bands migrated with molecular weights in agreement with that calculated for each fusion protein. Interestingly, Rgt2 and Snf3 appeared as discrete double and triple bands, respectively, suggesting posttranslational modifications of these transceptors. Because of the distinct character of the Rgt2 and Snf3 bands, posttranslational modifications like glycosylation and ubiquitylation may be excluded; rather phosphorylations may be suggested.

In brief, the results show that considerable amounts of partially purified transporter- and transceptor-fusion proteins can easily be isolated using a one-step block gradient procedure.

### Removal of GFP-8His tags using TEV protease

**Figure 7:**
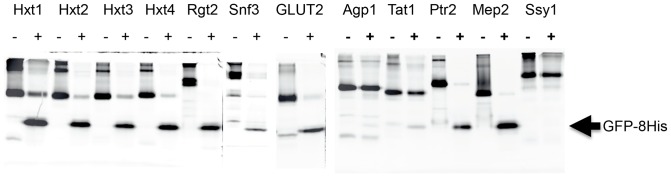
TEV protease digests of Ni-NTA purified transporter- and transceptor GFP-fusions. TEV protease digestions of the indicated GFP-fusions purified by Ni-NTA affinity chromatography. Digestions were analyzed by SDS-PAGE using in-gel fluorescence to visualize the GFP-fusions and the released GFP-8His. (−) undigested; (+) digested with TEV protease. Released GFP-8His is indicated with an arrow.

We analyzed the option to remove the GFP-8His tag from the fusion proteins by protease cleavage at a TEV site joining the membrane protein and the GFP-8-His tag (Figure 1). Undigested and TEV digested fusion proteins were analyzed by SDS-PAGE and in-gel fluorescence as shown in Figure 7. In the absence of TEV, intense protein bands corresponding to the full-length fusions are present on the gel; in addition, high molecular weight bands presumably representing oligomeric states like dimers and trimers are observed.

Exposure to TEV protease easily released the GFP-8His tag from the Hxt1-, Hxt2-, Hxt3-, Hxt4-, Rgt2-, Snf3-, GLUT2-, Ptr2-, and Mep2-fusions (Figure 7). However, the APC family transporter-fusions (Agp1-, Tat1- and Ssy1-fusions) appeared to be problematic. The Agp1- and Tat1 fusions were only slightly digested, and the Ssy1 fusion was completely resistant to TEV. Increasing the TEV:fusion ratio and digestion temperature resulted in cleavage of the Agp1 and Tat1 fusions; however, the Ssy1 fusion remained insensitive to TEV cleavage (not shown).

### Purification of Ptr2 by reverse immobilized metal-affinity chromatography

**Figure 8:**
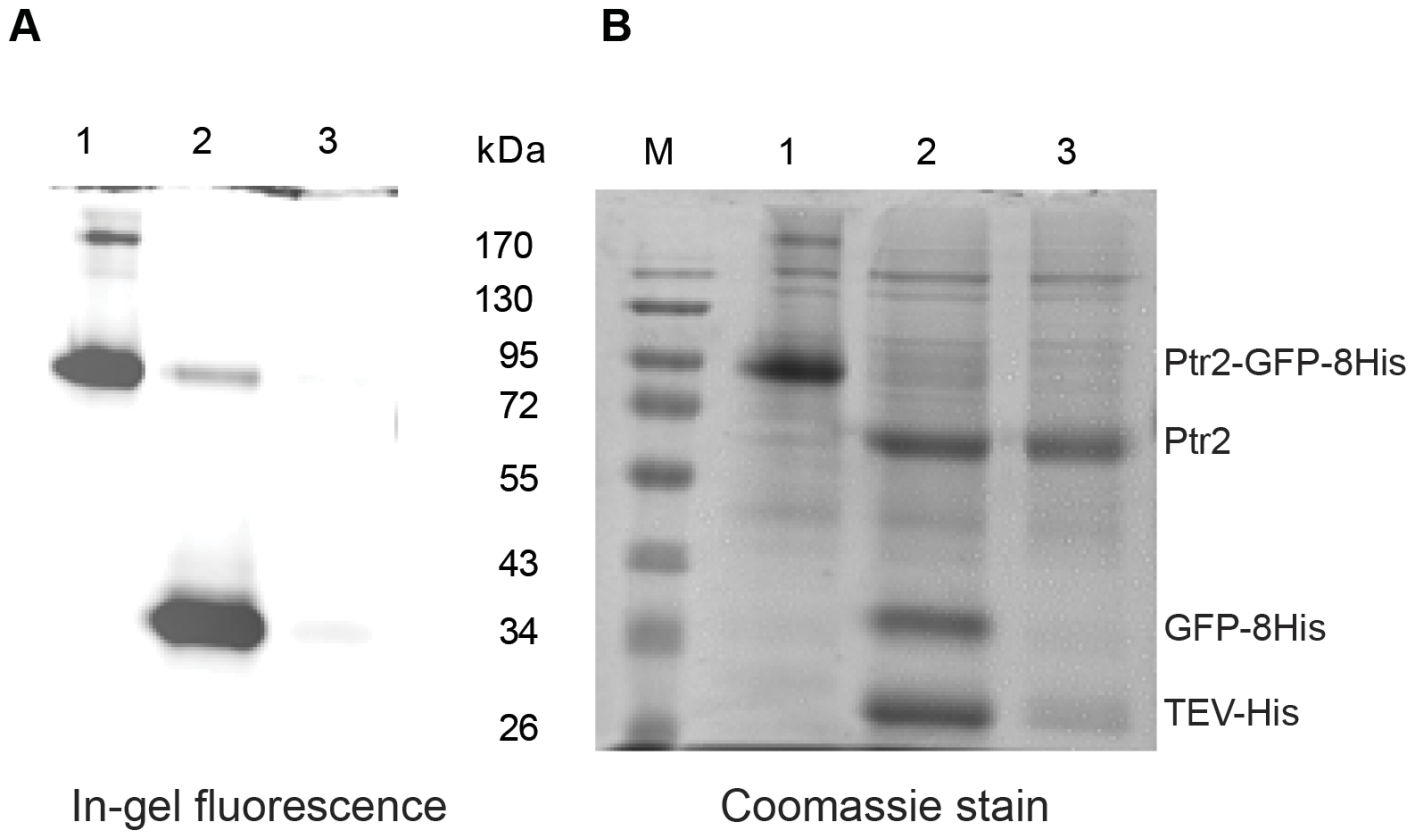
Purification of the Ptr2 transporter. Membranes from a 1 litre shake culture were solubilised in DDM and applied to a Ni-NTA column. Ptr2-GFP-His fusions were eluted from the column and digested with TEV protease. Purification of Ptr2 and removal of the released GFP-8His tag was carried out by reverse IMAC. Samples from the 3-step procedure were analysed by SDS-PAGE in a 10% gel. (A) Detection by in-gel fluorescence. (B) Detection by Coomassie staining. Lane 1: Ni-NTA elution. Lane 2: Cleavage by TEV protease. Lane 3: Flow-through of reverse Ni-NTA chromatography after cleavage with TEV protease. M: PageRuler prestained protein ladder (Fermentas). Positions of the Ptr2-GFP-His fusion, Ptr2 transporter, cleaved off GFP-8His and the TEV protease are indicated.

Ptr2 was further purified by reverse immobilized metal-affinity chromatography (IMAC) to remove the GFP-8His tag, the His-tagged TEV protease and co-eluting contaminating proteins by passing the TEV cleavage digestion through a Ni-NTA resin [19]. The results (Figure 8) show that an almost pure preparation of Ptr2 can be achieved by this procedure, yielding approximately 0.2 mg purified Ptr2 from a 1-liter shake culture.

These results indicate that large amounts of purified Ptr2 will be accessible using a 10-liter fermenter for propagation and induction of the expression strain. Similarly, we expect that additional eukaryotic transporters and transceptors may be produced for functional and structural analysis using the pipeline for expression and purification outlined in this article.

### Evaluation of the quality of GFP-fusions by FSEC

**Figure 9:**
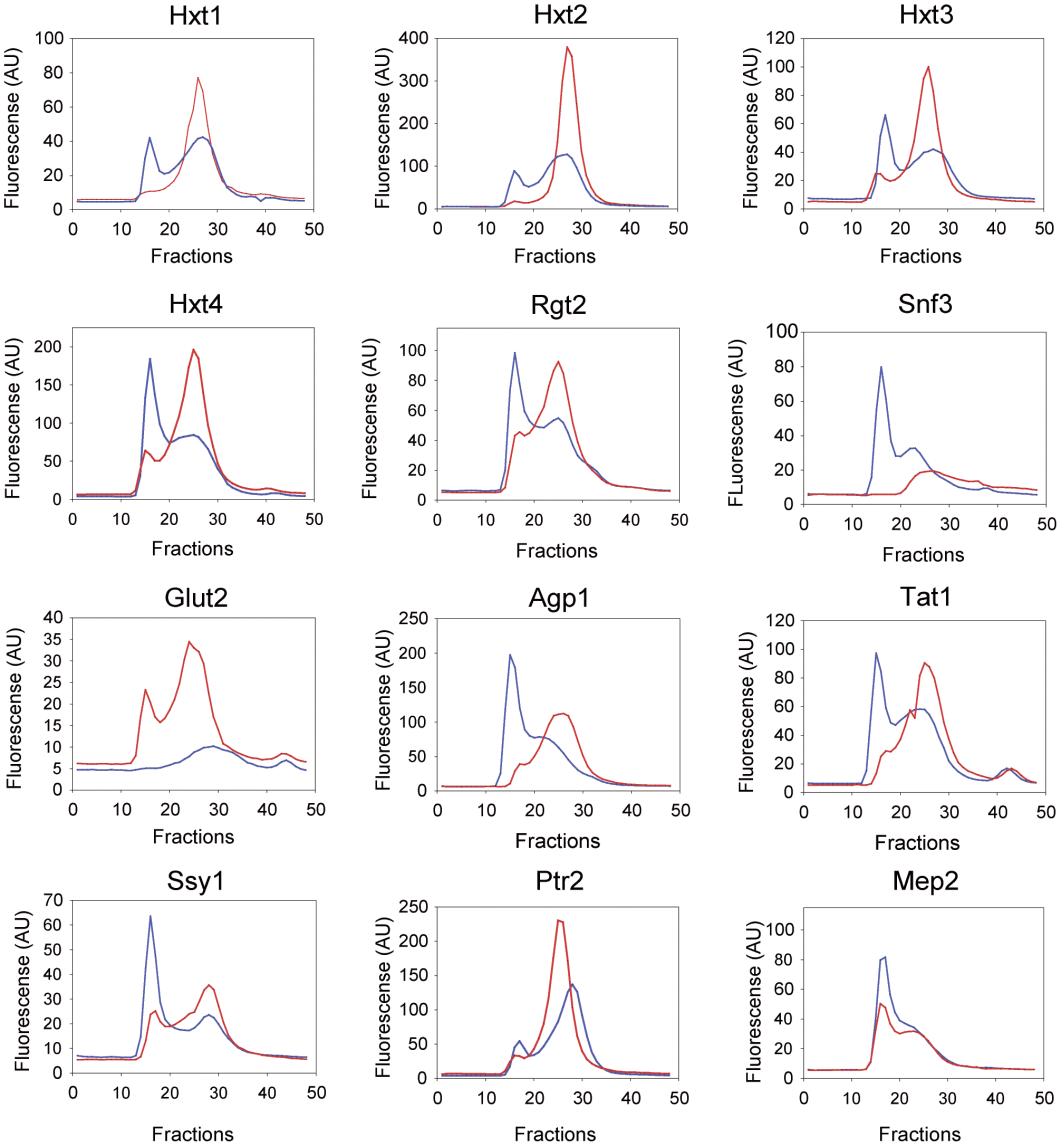
FSEC profiles of transporter and transceptor GFP-fusions. FSEC analysis of total membranes solubilized in DDM (blue line) or in DDM, CHS and specific substrates (red line). Membranes were isolated from yeast cells, which were induced for overexpression of the GFP-fusions at 15°C for 48 hours. The solubilized membranes were separated on a Superose 12 10/300 column. Fractions were collected and measured for their GFP fluorescence. Molecular weight markers (GE Healthcare Life Science) separated on the same column peaked as follows: Blue Dextran 2000, 2000 kDa, fraction 14, corresponding to a void volume of 7.6 ml; thyroglobulin, 669 kDa, fraction 15; ferritin, 440 kDa, fraction 23; aldolase, 158 kDa, fraction 32; conalbumin, 75 kDa, fraction 36; purified yEGFP (this work), 28 kDa, fraction 45.

Membranes containing transporter- and transceptor-GFP fusions were solubilized in DDM for assessment of the quality by FSEC [19], [25]. In general, FSEC elution profiles with two peaks were obtained (Figure 9). The first peak eluted in the void volume in fractions 14 to 19 with a peak top in fraction 16, and probably represents higher order aggregates. The second peak eluted in fractions 20 to 30 and most likely represents well-folded transporter/transceptor oligomers.

To reduce the content of aggregated proteins during solubilization, we performed extractions in a mixture of DDM, CHS and appropriate substrates. The resulting FSEC profiles (Figure 9) show that the Hxt1- and Hxt2-preparations were monodisperse, whereas the Hxt3-, Hxt4- and Rgt2-preparations only contained small amounts of aggregated protein. The content of aggregated fusions was also eliminated from the Snf3-preparation; however, the fusion eluted in a broad peak with a lagging shoulder. The GLUT2-fusion, which was poorly solubilized in DDM, was much more efficiently solubilized in DDM, CHS and glucose; however with a small amount of aggregated fusion protein.

The Agp1-, Tat1 and Ssy1-preparations were also improved by removal of a large fraction of aggregated protein. The profile of the Ptr2-fusion has a minor peak at the void volume followed by a major symmetric peak eluting in about 2 ml. For the Mep2-fusion a slight improvement was observed; i.e. a major peak at the void volume followed by a broad peak of potentially well-folded fusion protein.

The approximate molecular weights of the GFP-fusions in complex with DDM were assessed using the elution volumes of soluble-protein standards (see legend to Figure 9). According to the standards, the membrane protein-detergent complexes in the second peak have molecular weights in the range between 300 to 440 kDa. These estimates are probably exaggerated, since membrane protein-detergent complexes elute ahead of soluble-protein standards [36], suggesting that the complexes have molecular weights in the range of 200 to 300 kDa. Bearing in mind that the amount of detergent and CHS bound to the analyzed fusion proteins is unknown, a cautious approximation may suggest that the peaks represent oligodimers or oligotrimers, since the expected monomer size of the membrane protein-GFP fusions are in the range of 81 to 125 kDa (see legend to Figure 4).

## Discussion

The great challenges in structural studies of membrane proteins are evident from the very few membrane protein structures available in the database of Membrane Proteins of Known 3D Structure (http://blanco.biomol.uci.edu/mpstruc/listAll/list). Presently, the number of solved membrane protein structures in this database is in the range of 310, which is rather low considering the more than 91,000 structure entries of soluble proteins in the online Protein Data Bank.

At present, the list of structures of transporters of the MFS and of the APC superfamily in the database of Membrane Proteins of Known 3D Structure includes only 10 and 6 structures of prokaryotic origin, respectively, whereas no eukaryotic structures are yet available. Examples of crystal structures of bacterial members of the APC family comprise the arginine-dependent arginine:agmatine antiporter AdiC from *E. coli* [37], [38] and a broad-specificity amino acid transporter ApcT from *Methanocaldococcus jannaschii* [39]. Examples of crystal structures of bacterial members of the MFS comprise lactose permease from *E. coli* [40] and the PepT_So_ oligopeptide-proton symporter from *Shewanella oneidensis* [41].

As discussed in recent reports, the isolation of eukaryotic membrane proteins produced in *S. cerevisiae* is challenging, and the limited number of membrane protein structures is due to difficulties encountered with production, solubilization and purification of appropriate amounts of membrane proteins that are able to form crystals diffracting at a high resolution [42], [43]. To explore the possibilities of improving production of eukaryotic membrane proteins in *S. cerevisiae* we set forth to combine our know-how in a high-copy expression system [18] with contemporary membrane protein-GFP fusion technologies [19]. This initiative is motivated by our interest in understanding the molecular mechanisms of the yeast amino acid and glucose transceptors and their related amino acid and glucose transporters.

Due to our interest in nutrient transporters and transceptors we here made constructs that overexpress four hexose transporters (Hxt1, Hxt2, Hxt3 and Hxt4), two glucose transceptors (Snf3 and Rgt2), two amino acid transporters (Agp1 and Tat1), an amino acid transceptor (Ssy1), an oligo peptide transporter (Ptr2), and an ammonium transceptor (Mep2) from *S. cerevisiae* as fusions to GFP. To test expression of a mammalian transceptor we also included the human glucose transceptor GLUT2 in this study.

We found that the twelve membrane proteins were expressed as full-length GFP-fusions and located to the plasma membrane and/or intracellular membranes (Figure 3, Figure 4). As discussed in the results section some of the transporters and transceptors may accumulate in Golgi. This may be considered a draw-back leading to inactive transporters. However, as observed in [44] a plant plasma membrane H^+^-ATPase overexpressed in yeast was localized to the endoplasmatic reticulum and not the plasma membrane as expected; yet it was fully acticve. In accordance with this finding, we propose that the highly overexpressed transporters and transceptors produced in the present study challenges the capacity of the secretory pathway and leads to accumulation of functional fusions in intracellular membranes such as Golgi.

The expression vector used in this study has the advantages of a strong promoter, a very high-copy-number feature and selection markers that prevent plasmid-loss. Furthermore, the host strain PAP1500 is tailored to overproduce the Gal4 transcription factor simultaneously with induction of membrane protein production to maximize transcription from the extraordinary high number of plasmids.

Using this expression system we were able to produce the 12 targets in this study in the range of 0.4 mg to 1.7 mg transporter/transceptor pr. liter culture, when expression strains are grown in shake flasks (Table 1). The capacity of our expression system is high-lighted by the overexpression of the Ssy1-GFP fusion. We obtained a level of Ssy1-GFP of 0.5 mg/L cell culture (Table 1). In contrast, Ssy1 could not be detected in other studies [45], [46], and only very low expression was reported in [47]. Similarly, we were unable to detect any expression of Ssy1 using the pYES2 overexpression vector (Invitrogen) (unpuplished results). Obviously, the features of the PAP1500-GFP expression system make a huge difference for stable overexpression of Ssy1-GFP fusions. To our knowledge, the 12 transporters and transceptors in the present study have not previously been overexpressed to levels that allow purification. Presumeably, because yeast expression systems with an efficiency comparable to our system have not been available.

In the present study we decided to investigate production of the GFP-fusions in shake flask cultures induced for 48 hours at 15°C. As evident from the optimization experiments with two fusions in Figure 2, it is very likely that individual optimization of growth and induction conditions for each transporter tested in our expression system may improve the yield. For example the Ssy1-GFP-fusion accumulated stably at 15°C with a 48 hour induction, whereas the Agp1-GFP-His fusion peaked after 24 hours at 25°C. Furthermore, when production is scaled up in computer controlled fermenters, the yield will presumably be increased considerably according to our experience with the high-copy vector system [18].Thus, our induction protocol usually produce about 3 grams of wet weight cells in 1 liter shake cultures, whereas a 10-liter fermenter produces 250 grams. Taken together, we expect that the yield of membrane proteins may be increased in the range of 10 times after individual induction optimizations and production in fermenters.

We also report a fast detergent screen based on centrifugations in a Beckman airfuge, which reduces the consumption of membranes and detergent as well as the centrifugation time. Generally, we found that DDM, DM, FC-12 and CYMAL-5 solubilized the GFP fusions with an acceptable efficiency of about 50 to 100%, whereas OG and CHAPS gave poor solubilization (Figure 5). This screening method may be used in pre-crystallization screenings for selection of detergents that produce monodisperse and stable membrane proteins suitable for obtaining high-resolution X-ray structures.

The monodispersity of the GFP-fusions solubilized either in DDM or in DDM with CHS and specific substrates was analyzed by FSEC [19], [25]. For most of the fusions solubilization in DDM resulted in a high content of aggregated protein (Figure 9). However, solubilization in DDM supplemented with CHS and specific substrates resulted in highly improved FSEC profiles with small amounts of aggregated fusions (Figure 9). In particular, Hxt1-, Hxt2-, Hxt3-, Hxt4-, Rgt2-, GLUT2- and Ptr2-fusions gave preparations that can be used straight away for purification and crystallization trials. The remaining fusions need further screenings for improved profiles in more suitable detergents and panels of additives such as ligands and lipids. This may rescue aggregated proteins and give the desired monodisperse preparations. Alternatively, aggregated material may be resolved from the presumably well-folded soluble protein by preparative size exclusion chromatography to deliver monodisperse preparations ideal for crystallization trials. As discussed in for example [19], [42], [32], [48] monodisperse preparations are not easily obtained and often numerous optimization attempts must be carried out. However, using the PAP1500-GFP expression system with induction at 15°C for 48 hours a high degree of monodispersity was easily obtained for the majority of membrane proteins in this study. Thus, application of our expression system combined with low temperature induction conditions appears to be favorable for producing a variety of membrane proteins.

In the present study we demonstrate that partially purified transporter- and transceptor-GFP-fusions can easily be obtained from DDM solubilized membranes using a simple one-step Ni-NTA affinity procedure (Figure 6). Ni-NTA resin binding efficiencies were in the range of 70% and the recovery was on average 50%. To our knowledge, these results are the first reports on purification of these transporters and transceptors.

The purified Hxt1-, Hxt2-, Hxt3-, Hxt4-, Rgt2-, Snf3-, GLUT2-, Ptr2-, and Mep2-GFP-His fusions were readily cleaved by TEV protease for removal of the GFP-His tag (Figure 7). On the contrary, the Agp1- and Tat1-GFP fusions needed higher incubation temperatures for cleavage, whereas the Ssy1-GFP fusion was insensitive to cleavage at the employed conditions. Perhaps steric hindrance in the three APC family-GFP fusions makes the TEV protease site less accessible.

An example of successful removal of the GFP-tag from the transporter by TEV cleavage and reverse immobilized metal-affinity chromatography (IMAC) is shown for the Ptr2-GFP fusion (Figure 8). Using this approach approximately 0.2 mg purified Ptr2 was obtained from a 1-liter culture. Following this purification scheme, similar results will most likely be achievable for the other TEV cleavable GFP-fusions in this study; except for the APC family-GFP fusions, for which an alternative approach for proteolytic removal of the GFP tag must be explored.

Interestingly, the glucose transceptors Rgt2 and Snf3 migrated as double and triple bands, respectively, in SDS-PAGE analysis (Figure 6). These observations have not been reported previously, and may suggest posttranslational modifications such as phosphorylations active in sensing or trafficking mechanisms [49] during the high-galactose induction conditions.

Taken together, we show that the PAP1500-GFP expression system has the potential to deliver mg amounts of endogenous yeast nutrient transceptors and transporters with acceptable FSEC profiles. We have recently published the use of our expression system for production of human Aquaporin-1, which accumulated in unexpected high levels constituting 8.5% of the total membrane protein content [50]. Similarly, we were able to produce high quality of rat Adenosine A1 receptor in our system yielding 1.9% of the total membrane protein content (unpublished data). With these results, we demonstrate that application of the PAP1500-GFP expression system and low temperature induction conditions may facilitate production of high amounts of a wide variety of eukaryotic membrane proteins in a quality that pave the way for biophysical, functional and structural studies.

## Supporting Information

Table S1 Strains used in this study.
(DOCX)

Table S2 Primers used in this study. Nucleotide sequences shown in bold are complementary to the template. Human GLUT2 was amplified from a plasmid with GLUT2 cDNA obtained from Dr. Armelle Leturque. The sequence shown in italics is the Kozak sequence from the yeast PMR1 gene used for GLUT2. All other sequences are used for homologous recombination.
(DOCX)
