# Supplementary material for: Saccharomyces cerevisiae–Based Platform for Rapid Production and Evaluation of Eukaryotic Nutrient Transporters and Transceptors for Biochemical Studies and Crystallography
Source: PLoS One. 2013 Oct 4;8(10):e76851. doi: 10.1371/journal.pone.0076851 (PMC3790737; doi:10.1371/journal.pone.0076851)
Supplement: Table S1 — Strains used in this study. (DOCX) [file pone.0076851.s001.docx]

Table S1

| ***S. cerevisiae* strains** | **Genotype** | **Reference** |
| --- | --- | --- |
| PAP1500 | *MATα* *ura3*-*52 trp1:: GAL10-GAL4 lys2*-*801 leu2*Δ*1 his3*Δ*200 pep4::HIS3 prb1*Δ*1.6R can1 GAL* | [Pedersen et al 1996)] |
| PAP7910 | PAP1500/pSsy1-GFP-8His | This work |
| PAP8004 | PAP1500/pAgp1-GFP-8His | This work |
| PAP8139 | PAP1500/pMep2-GFP-His | This work |
| PAP8141 | PAP1500/pPtr2-GFP-His | This work |
| PAP8143 | PAP1500/pTat1-GFP-His | This work |
| PAP8131 | PAP1500/pHxt1-GFP-His | This work |
| PAP8133 | PAP1500/pHxt2-GFP-His | This work |
| PAP8135 | PAP1500/pHxt3-GFP-His | This work |
| PAP8137 | PAP1500/pHxt4-GFP-His | This work |
| PAP8145 | PAP1500/pRgt2-GFP-His | This work |
| PAP8147 | PAP1500/pSnf3-GFP-His | This work |
| PAP7913 | PAP1500/pGLUT2-GFP-His | This work |
| ***E. coli* strains** |  |  |
| BL21(DE3)pLysS | F- *ompT hsdS*B (rB^-^mB^-^) *gal dcm* (DE3) pLysS (Cam^R^) | Invitrogen |
| BL21(DE3) Codon Plus RIL | B F– *ompT hsdS*(rB^–^ mB^–^) *dcm*^+^ Tetr *gal* λ(DE3) *endA* Hte [*argU ileYleuW* Cam^R^] | Stratagene |
| PAP7841 | BL21(DE3)pLysS/ pET20bGFP-8His Amp^R^ | This work |
| PAP8350 | BL21(DE3) Codon Plus RIL/pMBP-TEV Amp^R^ | This work |
