# Supplementary material for: Saccharomyces cerevisiae–Based Platform for Rapid Production and Evaluation of Eukaryotic Nutrient Transporters and Transceptors for Biochemical Studies and Crystallography
Source: PLoS One. 2013 Oct 4;8(10):e76851. doi: 10.1371/journal.pone.0076851 (PMC3790737; doi:10.1371/journal.pone.0076851)
Supplement: Table S2 — Primers used in this study. Nucleotide sequences shown in bold are complementary to the template. Human GLUT2 was amplified from a plasmid with GLUT2 cDNA obtained from Dr. Armelle Leturque. The sequence shown in italics is the Kozak sequence from the yeast PMR1 gene used for GLUT2. All other sequences are used for homologous recombination. (DOCX) [file pone.0076851.s002.docx]

Table S2

| Primer | DNA sequence 5’🡺 3’ |
| --- | --- |
| GFPup | GAAAATTTGTATTTTCAAAGTCAATTT**TCTAAAGGTGAAGAATTATTCACT** |
| GFPHISdo | CTTCAATGCTATCATTTCCTTTGATATTGGATCATCTAATGGTGATGGTGATGGTGATGGTG**TTTGTACAATTCATCCATACCAT** |
| SSY1up | ACACAAATACACACACTAAATTACCGATCAATTC**CTTGAGGAATATGAGTTCT** |
| SSY1TEVdo | AAATTGACTTTGAAAATACAAATTTTC**AATCAGCCAGGTAACCAACT** |
| TAT1up | ACACAAATACACACACTAAATTACCGGATCAATT**CGGCGTAAAAATGGACGATAGT** |
| TAT1TEVdo | AAATTGACTTTGAAAATACAAATTTTCGCACCAGAAATTGGTCATCC |
| MEP2TEVup | ACACAAATACACACACTAAATTACCGGATCAATT**CCAACAATGTCTTACAATTTTACAGG** |
| MEP2TEVdo | AAATTGACTTTGAAAATACAAATTTTC**TACTATATGGTCAGTGTTCTTAG** |
| CAN1up | ACACAAATACACACACTAAATTACCGGATCAATT**CAGGCATAGCAATGACAAATTC** |
| CAN1TEVdo | AAATTGACTTTGAAAATACAAATTTTCTGCTACAACATTCCAAAATTTG |
| PTR2up | ACACAAATACACACACTAAATTACCGATCAATT**CCAATATCAATATGCTCAACCATCCCAGCC** |
| PTR2TEVdo | AAATTGACTTTGAAAATACAAATTTTC**ATATTTGGTGGTGGATCTTAGA** |
| HXT1up | ACACAAATACACACACTAAATTACCGATCAATTCACGTAAAATCATGAATTCAACT |
| HXT1TEVdo | AAATTGACTTTGAAAATACAAATTTTCTTTCCTGCTAAACAAACTCTT |
| HXT2up | ACACAAATACACACACTAAATTACCGATCAATTCAAGCAACATAATGTCTGAATTC |
| HXT2TEVdo | AAATTGACTTTGAAAATACAAATTTTCTTCCTCGGAAACTCTTTTTTC |
| HXT3up | ACACAAATACACACACTAAATTACCGATCAATTCTTAAACAATCATGAATTCA |
| HXT3TEVdo | AAATTGACTTTGAAAATACAAATTTTCTTTCTTGCCGAACATTTTCTTG |
| HXT4up | ACACAAATACACACACTAAATTACCGATCAATTCTCTGCCAAAAATGTCTGAA |
| HXT4TEVdo | AAATTGACTTTGAAAATACAAATTTTCCTTTTTTCCGAACATCTTCTTG |
| SNF3up | ACACAAATACACACACTAAATTACCGGATCAATT**CGAATTTATAAATGGATCCTAATAGT** |
| SNF3TEVdo | AAATTGACTTTGAAAATACAAATTTTC**TTTCAAATCATTATTTTCATTTACAG** |
| RGT2up | ACACAAATACACACACTAAATTACCGGATCAATT**CTATTGCTTGTATGAACGATAGC** |
| RGT2TEVdo | AAATTGACTTTGAAAATACAAATTTTC**TTGGGGGGAAGTGTATTGG** |
| AGP1up | ACACAAATACACACACTAAATTACCGGATCAATTCC**GCTTCGCACAATGTCGTC** |
| AGP1TEVdo | AAATTGACTTTGAAAATACAAATTTTC**ACACCAGAAGGCAACGAC** |
| GLUT2up | ACACAAATACACACACTAAATTACCGGATCAATT*CCTTGAGGAAT*ATGACAGAAGATAAGGTCACT |
| GLUT2TEVdo | AAATTGACTTTGAAAATACAAATTTTCCACAGTCTCTGTAGCTCCT |
